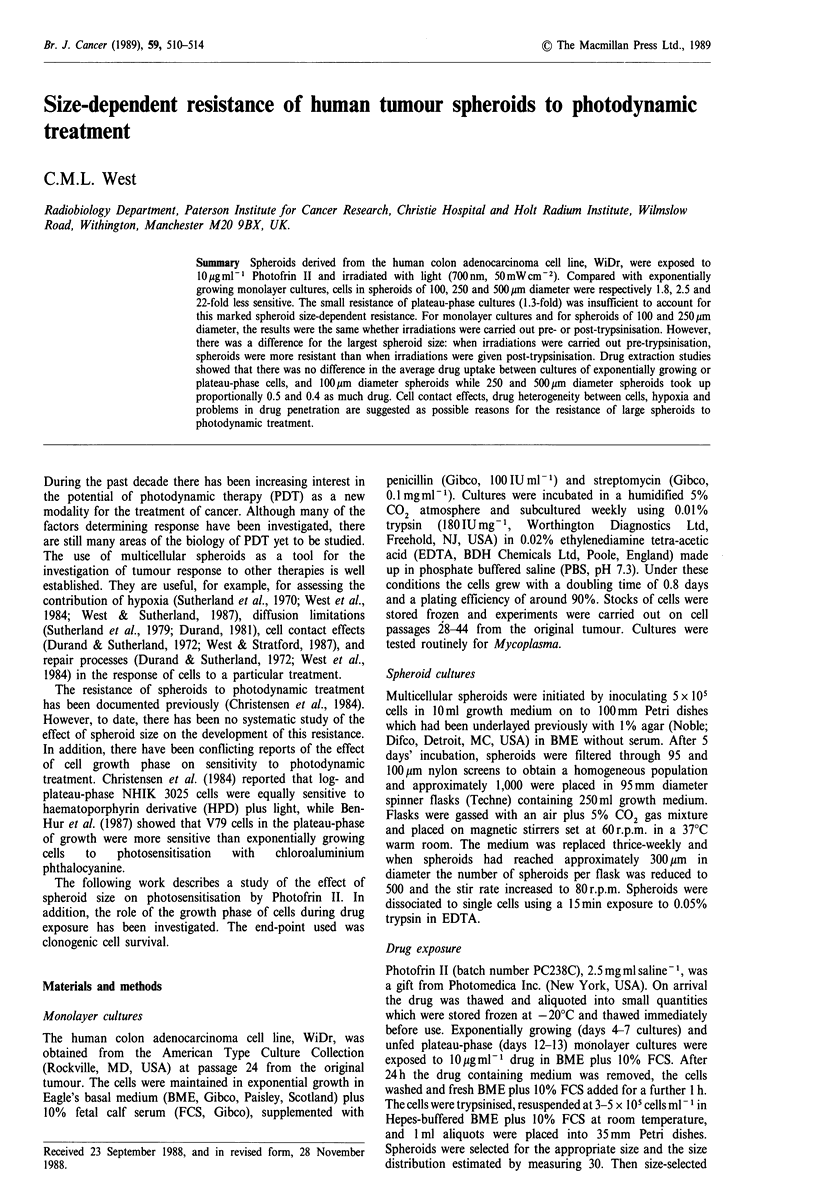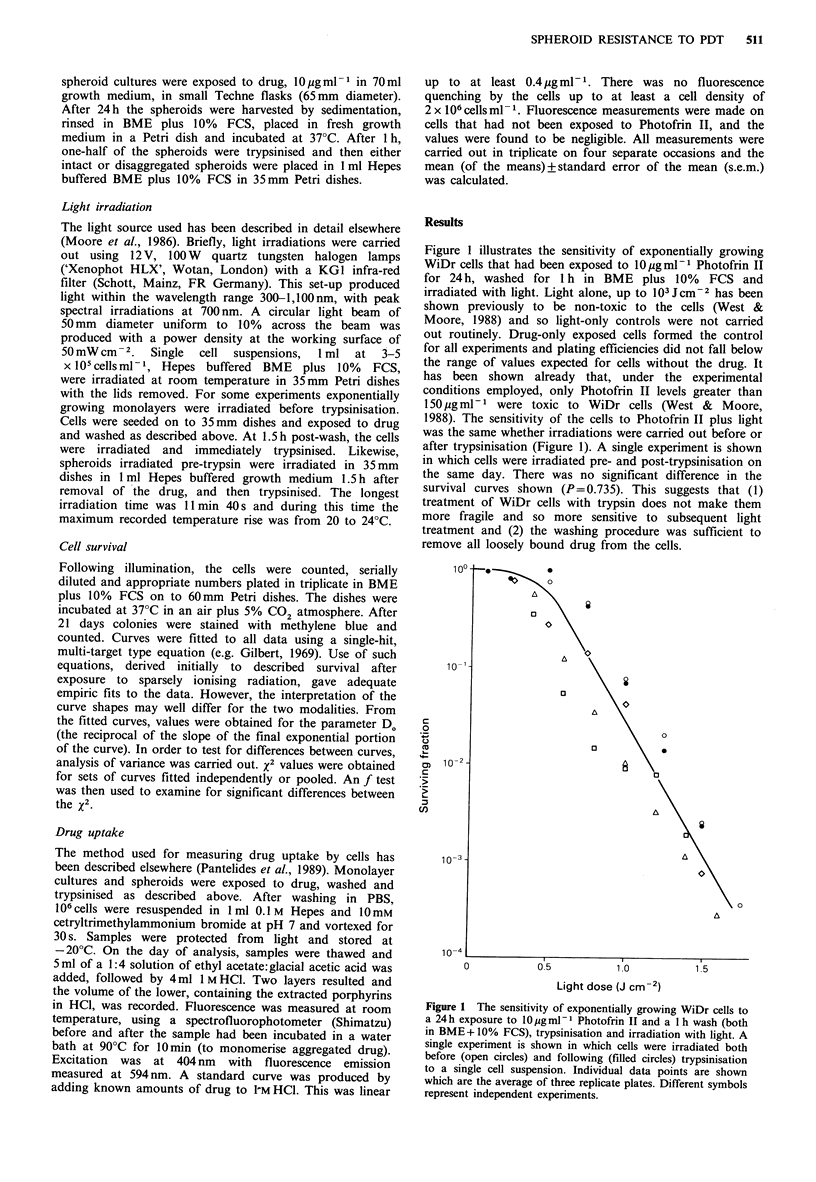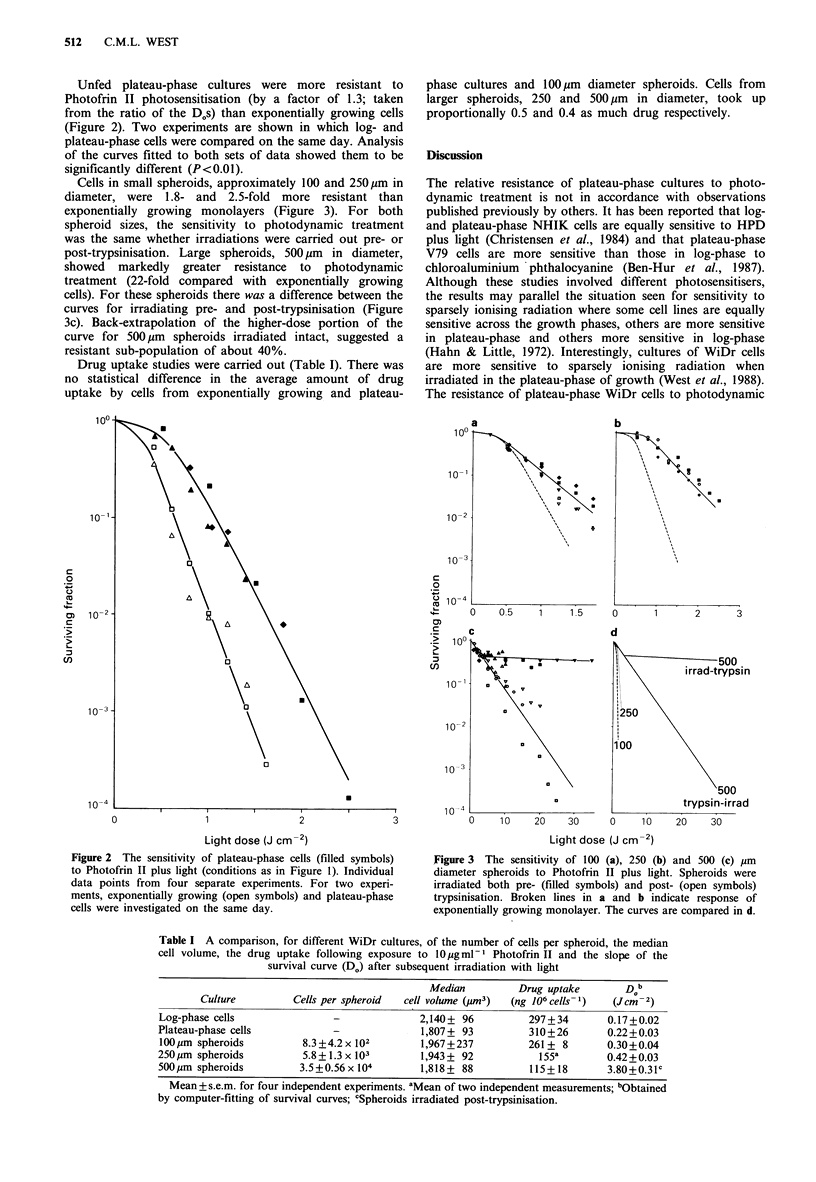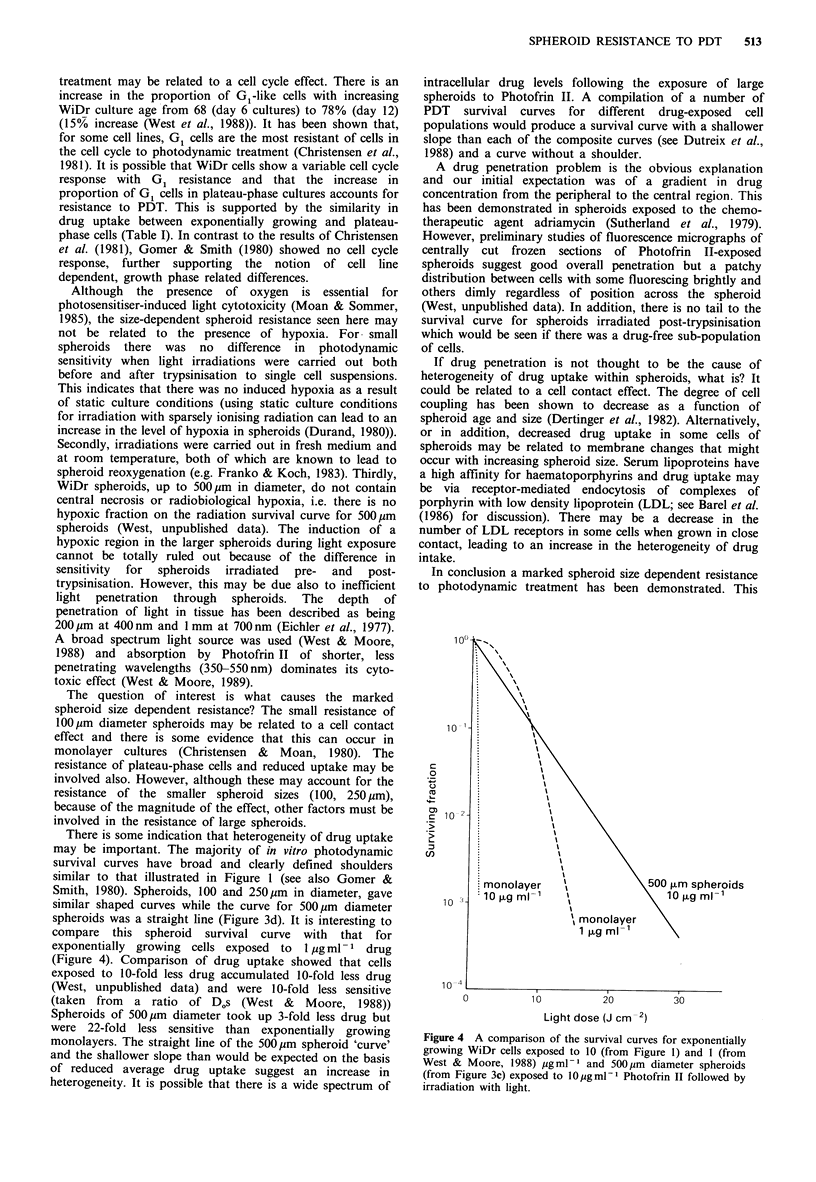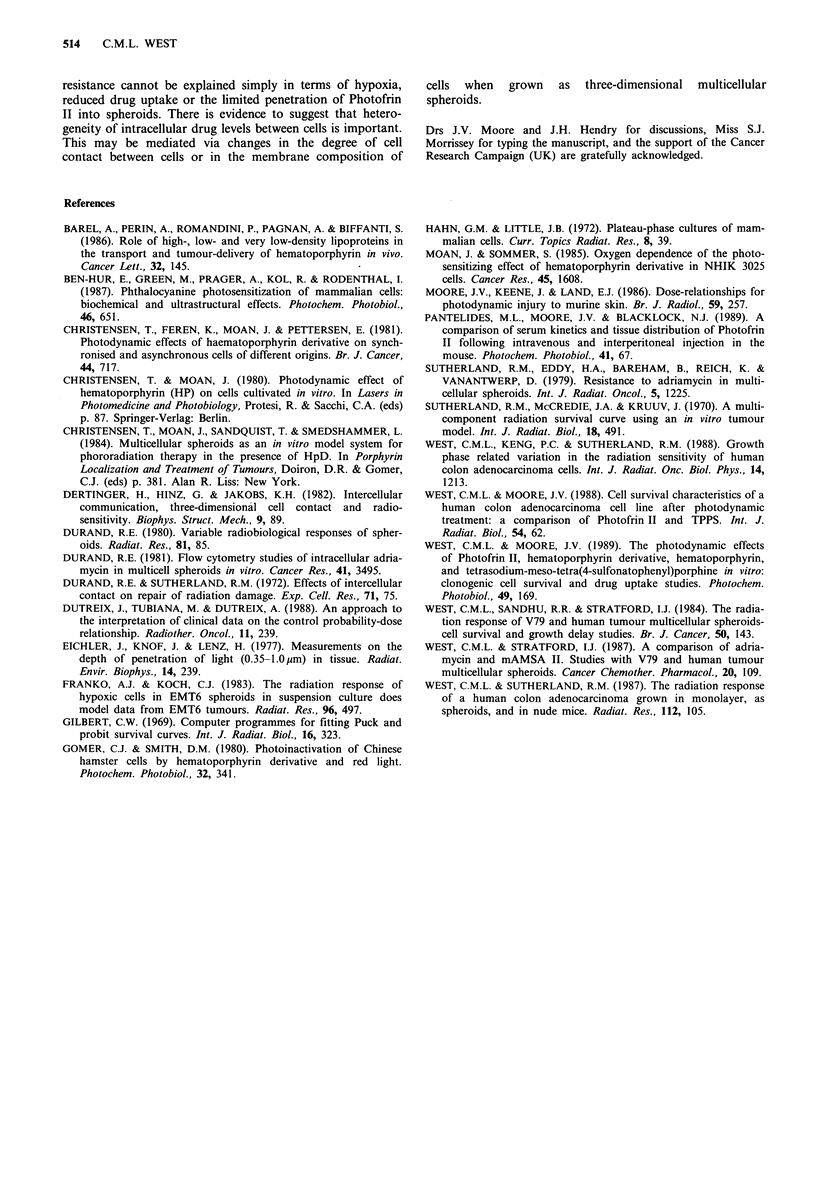# Size-dependent resistance of human tumour spheroids to photodynamic treatment.

**DOI:** 10.1038/bjc.1989.105

**Published:** 1989-04

**Authors:** C. M. West

**Affiliations:** Radiobiology Department, Paterson Institute for Cancer Research, Christie Hospital and Holt Radium Institute, Manchester, UK.

## Abstract

Spheroids derived from the human colon adenocarcinoma cell line, WiDr, were exposed to 10 micrograms ml-1 Photofrin II and irradiated with light (700 nm, 50 mW cm-2). Compared with exponentially growing monolayer cultures, cells in spheroids of 100, 250 and 500 microns diameter were respectively 1.8, 2.5 and 22-fold less sensitive. The small resistance of plateau-phase cultures (1.3-fold) was insufficient to account for this marked spheroid size-dependent resistance. For monolayer cultures and for spheroids of 100 and 250 microns diameter, the results were the same whether irradiations were carried out pre- or post-trypsinisation. However, there was a difference for the largest spheroid size: when irradiations were carried out pre-trypsinisation, spheroids were more resistant than when irradiations were given post-trypsinisation. Drug extraction studies showed that there was no difference in the average drug uptake between cultures of exponentially growing or plateau-phase cells, and 100 microns diameter spheroids while 250 and 500 microns diameter spheroids took up proportionally 0.5 and 0.4 as much drug. Cell contact effects, drug heterogeneity between cells, hypoxia and problems in drug penetration are suggested as possible reasons for the resistance of large spheroids to photodynamic treatment.


 © The Macmillan Press Ltd., 1989

### Size-dependent resistance of human tumour spheroids to photodynamic treatment

C.M.L. West

*Radiobiology Department, Paterson Institute for Cancer Research, Christie Hospital and Holt Radium Institute, Wilmslow Road, Withington, Manchester M20 9BX, UK.*

**Summary** Spheroids derived from the human colon adenocarcinoma cell line, WiDr, were exposed to $10\,\mu g\,ml^{-1}$ Photofrin II and irradiated with light (700 nm, $50\,mW\,cm^{-2}$). Compared with exponentially growing monolayer cultures, cells in spheroids of 100, 250 and 500 $\mu m$ diameter were respectively 1.8, 2.5 and 22-fold less sensitive. The small resistance of plateau-phase cultures (1.3-fold) was insufficient to account for this marked spheroid size-dependent resistance. For monolayer cultures and for spheroids of 100 and 250 $\mu m$ diameter, the results were the same whether irradiations were carried out pre- or post-trypsinisation. However, there was a difference for the largest spheroid size: when irradiations were carried out pre-trypsinisation, spheroids were more resistant than when irradiations were given post-trypsinisation. Drug extraction studies showed that there was no difference in the average drug uptake between cultures of exponentially growing or plateau-phase cells, and 100 $\mu m$ diameter spheroids while 250 and 500 $\mu m$ diameter spheroids took up proportionally 0.5 and 0.4 as much drug. Cell contact effects, drug heterogeneity between cells, hypoxia and problems in drug penetration are suggested as possible reasons for the resistance of large spheroids to photodynamic treatment.

During the past decade there has been increasing interest in the potential of photodynamic therapy (PDT) as a new modality for the treatment of cancer. Although many of the factors determining response have been investigated, there are still many areas of the biology of PDT yet to be studied. The use of multicellular spheroids as a tool for the investigation of tumour response to other therapies is well established. They are useful, for example, for assessing the contribution of hypoxia (Sutherland *et al.*, 1970; West *et al.*, 1984; West & Sutherland, 1987), diffusion limitations (Sutherland *et al.*, 1979; Durand, 1981), cell contact effects (Durand & Sutherland, 1972; West & Stratford, 1987), and repair processes (Durand & Sutherland, 1972; West *et al.*, 1984) in the response of cells to a particular treatment.

The resistance of spheroids to photodynamic treatment has been documented previously (Christensen *et al.*, 1984). However, to date, there has been no systematic study of the effect of spheroid size on the development of this resistance. In addition, there have been conflicting reports of the effect of cell growth phase on sensitivity to photodynamic treatment. Christensen *et al.* (1984) reported that log- and plateau-phase NHIK 3025 cells were equally sensitive to haematoporphyrin derivative (HPD) plus light, while Ben-Hur *et al.* (1987) showed that V79 cells in the plateau-phase of growth were more sensitive than exponentially growing cells to photosensitisation with chloroaluminium phthalocyanine.

The following work describes a study of the effect of spheroid size on photosensitisation by Photofrin II. In addition, the role of the growth phase of cells during drug exposure has been investigated. The end-point used was clonogenic cell survival.

### Materials and methods

### *Monolayer cultures*

The human colon adenocarcinoma cell line, WiDr, was obtained from the American Type Culture Collection (Rockville, MD, USA) at passage 24 from the original tumour. The cells were maintained in exponential growth in Eagle's basal medium (BME, Gibco, Paisley, Scotland) plus 10% fetal calf serum (FCS, Gibco), supplemented with penicillin (Gibco, $100\,IU\,ml^{-1}$) and streptomycin (Gibco, $0.1\,mg\,ml^{-1}$). Cultures were incubated in a humidified 5% $CO_2$ atmosphere and subcultured weekly using 0.01% trypsin ($180\,IU\,mg^{-1}$, Worthington Diagnostics Ltd, Freehold, NJ, USA) in 0.02% ethylenediamine tetra-acetic acid (EDTA, BDH Chemicals Ltd, Poole, England) made up in phosphate buffered saline (PBS, pH 7.3). Under these conditions the cells grew with a doubling time of 0.8 days and a plating efficiency of around 90%. Stocks of cells were stored frozen and experiments were carried out on cell passages 28–44 from the original tumour. Cultures were tested routinely for *Mycoplasma*.

### *Spheroid cultures*

Multicellular spheroids were initiated by inoculating $5 \times 10^5$ cells in 10 ml growth medium on to 100 mm Petri dishes which had been underlayed previously with 1% agar (Noble; Difco, Detroit, MC, USA) in BME without serum. After 5 days' incubation, spheroids were filtered through 95 and 100 $\mu m$ nylon screens to obtain a homogeneous population and approximately 1,000 were placed in 95 mm diameter spinner flasks (Techne) containing 250 ml growth medium. Flasks were gassed with an air plus 5% $CO_2$ gas mixture and placed on magnetic stirrers set at 60 r.p.m. in a 37°C warm room. The medium was replaced thrice-weekly and when spheroids had reached approximately 300 $\mu m$ in diameter the number of spheroids per flask was reduced to 500 and the stir rate increased to 80 r.p.m. Spheroids were dissociated to single cells using a 15 min exposure to 0.05% trypsin in EDTA.

### *Drug exposure*

Photofrin II (batch number PC238C), $2.5\,mg\,ml\,saline^{-1}$, was a gift from Photomedica Inc. (New York, USA). On arrival the drug was thawed and aliquoted into small quantities which were stored frozen at −20°C and thawed immediately before use. Exponentially growing (days 4–7 cultures) and unfed plateau-phase (days 12–13) monolayer cultures were exposed to $10\,\mu g\,ml^{-1}$ drug in BME plus 10% FCS. After 24 h the drug containing medium was removed, the cells washed and fresh BME plus 10% FCS added for a further 1 h. The cells were trypsinised, resuspended at $3–5 \times 10^5$ cells $ml^{-1}$ in Hepes-buffered BME plus 10% FCS at room temperature, and 1 ml aliquots were placed into 35 mm Petri dishes. Spheroids were selected for the appropriate size and the size distribution estimated by measuring 30. Then size-selected

Received 23 September 1988, and in revised form, 28 November 1988.

spheroid cultures were exposed to drug, $10\mu g\,ml^{-1}$ in 70 ml growth medium, in small Techne flasks (65 mm diameter). After 24 h the spheroids were harvested by sedimentation, rinsed in BME plus 10% FCS, placed in fresh growth medium in a Petri dish and incubated at 37°C. After 1 h, one-half of the spheroids were trypsinised and then either intact or disaggregated spheroids were placed in 1 ml Hepes buffered BME plus 10% FCS in 35 mm Petri dishes.

*Light irradiation*

The light source used has been described in detail elsewhere (Moore *et al.*, 1986). Briefly, light irradiations were carried out using 12 V, 100 W quartz tungsten halogen lamps ('Xenophot HLX', Wotan, London) with a KG1 infra-red filter (Schott, Mainz, FR Germany). This set-up produced light within the wavelength range 300–1,100 nm, with peak spectral irradiations at 700 nm. A circular light beam of 50 mm diameter uniform to 10% across the beam was produced with a power density at the working surface of $50\,mW\,cm^{-2}$. Single cell suspensions, 1 ml at 3–5 $\times 10^5\,cells\,ml^{-1}$, Hepes buffered BME plus 10% FCS, were irradiated at room temperature in 35 mm Petri dishes with the lids removed. For some experiments exponentially growing monolayers were irradiated before trypsinisation. Cells were seeded on to 35 mm dishes and exposed to drug and washed as described above. At 1.5 h post-wash, the cells were irradiated and immediately trypsinised. Likewise, spheroids irradiated pre-trypsin were irradiated in 35 mm dishes in 1 ml Hepes buffered growth medium 1.5 h after removal of the drug, and then trypsinised. The longest irradiation time was 11 min 40 s and during this time the maximum recorded temperature rise was from 20 to 24°C.

*Cell survival*

Following illumination, the cells were counted, serially diluted and appropriate numbers plated in triplicate in BME plus 10% FCS on to 60 mm Petri dishes. The dishes were incubated at 37°C in an air plus 5% $CO_2$ atmosphere. After 21 days colonies were stained with methylene blue and counted. Curves were fitted to all data using a single-hit, multi-target type equation (e.g. Gilbert, 1969). Use of such equations, derived initially to described survival after exposure to sparsely ionising radiation, gave adequate empiric fits to the data. However, the interpretation of the curve shapes may well differ for the two modalities. From the fitted curves, values were obtained for the parameter $D_o$ (the reciprocal of the slope of the final exponential portion of the curve). In order to test for differences between curves, analysis of variance was carried out. $\chi^2$ values were obtained for sets of curves fitted independently or pooled. An $f$ test was then used to examine for significant differences between the $\chi^2$.

*Drug uptake*

The method used for measuring drug uptake by cells has been described elsewhere (Pantelides *et al.*, 1989). Monolayer cultures and spheroids were exposed to drug, washed and trypsinised as described above. After washing in PBS, $10^6$ cells were resuspended in 1 ml 0.1 M Hepes and 10 mM cetryltrimethylammonium bromide at pH 7 and vortexed for 30 s. Samples were protected from light and stored at −20°C. On the day of analysis, samples were thawed and 5 ml of a 1:4 solution of ethyl acetate:glacial acetic acid was added, followed by 4 ml 1 M HCl. Two layers resulted and the volume of the lower, containing the extracted porphyrins in HCl, was recorded. Fluorescence was measured at room temperature, using a spectrofluorophotometer (Shimatzu) before and after the sample had been incubated in a water bath at 90°C for 10 min (to monomerise aggregated drug). Excitation was at 404 nm with fluorescence emission measured at 594 nm. A standard curve was produced by adding known amounts of drug to 1 M HCl. This was linear up to at least $0.4\mu g\,ml^{-1}$. There was no fluorescence quenching by the cells up to at least a cell density of $2\times10^6\,cells\,ml^{-1}$. Fluorescence measurements were made on cells that had not been exposed to Photofrin II, and the values were found to be negligible. All measurements were carried out in triplicate on four separate occasions and the mean (of the means) ± standard error of the mean (s.e.m.) was calculated.

### Results

Figure 1 illustrates the sensitivity of exponentially growing WiDr cells that had been exposed to $10\mu g\,ml^{-1}$ Photofrin II for 24 h, washed for 1 h in BME plus 10% FCS and irradiated with light. Light alone, up to $10^3\,J\,cm^{-2}$ has been shown previously to be non-toxic to the cells (West & Moore, 1988) and so light-only controls were not carried out routinely. Drug-only exposed cells formed the control for all experiments and plating efficiencies did not fall below the range of values expected for cells without the drug. It has been shown already that, under the experimental conditions employed, only Photofrin II levels greater than $150\mu g\,ml^{-1}$ were toxic to WiDr cells (West & Moore, 1988). The sensitivity of the cells to Photofrin II plus light was the same whether irradiations were carried out before or after trypsinisation (Figure 1). A single experiment is shown in which cells were irradiated pre- and post-trypsinisation on the same day. There was no significant difference in the survival curves shown ($P=0.735$). This suggests that (1) treatment of WiDr cells with trypsin does not make them more fragile and so more sensitive to subsequent light treatment and (2) the washing procedure was sufficient to remove all loosely bound drug from the cells.

**Figure 1** The sensitivity of exponentially growing WiDr cells to a 24 h exposure to $10\mu g\,ml^{-1}$ Photofrin II and a 1 h wash (both in BME + 10% FCS), trypsinisation and irradiation with light. A single experiment is shown in which cells were irradiated both before (open circles) and following (filled circles) trypsinisation to a single cell suspension. Individual data points are shown which are the average of three replicate plates. Different symbols represent independent experiments.

Unfed plateau-phase cultures were more resistant to Photofrin II photosensitisation (by a factor of 1.3; taken from the ratio of the $D_0$s) than exponentially growing cells (Figure 2). Two experiments are shown in which log- and plateau-phase cells were compared on the same day. Analysis of the curves fitted to both sets of data showed them to be significantly different ($P<0.01$).

Cells in small spheroids, approximately 100 and 250 $\mu$m in diameter, were 1.8- and 2.5-fold more resistant than exponentially growing monolayers (Figure 3). For both spheroid sizes, the sensitivity to photodynamic treatment was the same whether irradiations were carried out pre- or post-trypsinisation. Large spheroids, 500 $\mu$m in diameter, showed markedly greater resistance to photodynamic treatment (22-fold compared with exponentially growing cells). For these spheroids there *was* a difference between the curves for irradiating pre- and post-trypsinisation (Figure 3c). Back-extrapolation of the higher-dose portion of the curve for 500 $\mu$m spheroids irradiated intact, suggested a resistant sub-population of about 40%.

Drug uptake studies were carried out (Table I). There was no statistical difference in the average amount of drug uptake by cells from exponentially growing and plateau-phase cultures and 100 $\mu$m diameter spheroids. Cells from larger spheroids, 250 and 500 $\mu$m in diameter, took up proportionally 0.5 and 0.4 as much drug respectively.

### Discussion

The relative resistance of plateau-phase cultures to photodynamic treatment is not in accordance with observations published previously by others. It has been reported that log- and plateau-phase NHIK cells are equally sensitive to HPD plus light (Christensen *et al.*, 1984) and that plateau-phase V79 cells are more sensitive than those in log-phase to chloroaluminium phthalocyanine (Ben-Hur *et al.*, 1987). Although these studies involved different photosensitisers, the results may parallel the situation seen for sensitivity to sparsely ionising radiation where some cell lines are equally sensitive across the growth phases, others are more sensitive in plateau-phase and others more sensitive in log-phase (Hahn & Little, 1972). Interestingly, cultures of WiDr cells are more sensitive to sparsely ionising radiation when irradiated in the plateau-phase of growth (West *et al.*, 1988). The resistance of plateau-phase WiDr cells to photodynamic

**Figure 2** The sensitivity of plateau-phase cells (filled symbols) to Photofrin II plus light (conditions as in Figure 1). Individual data points from four separate experiments. For two experiments, exponentially growing (open symbols) and plateau-phase cells were investigated on the same day.

**Figure 3** The sensitivity of 100 (**a**), 250 (**b**) and 500 (**c**) $\mu$m diameter spheroids to Photofrin II plus light. Spheroids were irradiated both pre- (filled symbols) and post- (open symbols) trypsinisation. Broken lines in **a** and **b** indicate response of exponentially growing monolayer. The curves are compared in **d**.

**Table I** A comparison, for different WiDr cultures, of the number of cells per spheroid, the median cell volume, the drug uptake following exposure to 10 $\mu$g ml$^{-1}$ Photofrin II and the slope of the survival curve ($D_0$) after subsequent irradiation with light

| *Culture* | *Cells per spheroid* | *Median cell volume* ($\mu m^3$) | *Drug uptake* (*ng* $10^6$ *cells*$^{-1}$) | $D_0$[b] ($J\,cm^{-2}$) |
|---|---|---|---|---|
| Log-phase cells | – | 2,140 ± 96 | 297 ± 34 | 0.17 ± 0.02 |
| Plateau-phase cells | – | 1,807 ± 93 | 310 ± 26 | 0.22 ± 0.03 |
| 100 $\mu$m spheroids | $8.3 \pm 4.2 \times 10^2$ | 1,967 ± 237 | 261 ± 8 | 0.30 ± 0.04 |
| 250 $\mu$m spheroids | $5.8 \pm 1.3 \times 10^3$ | 1,943 ± 92 | 155[a] | 0.42 ± 0.03 |
| 500 $\mu$m spheroids | $3.5 \pm 0.56 \times 10^4$ | 1,818 ± 88 | 115 ± 18 | 3.80 ± 0.31[c] |

Mean ± s.e.m. for four independent experiments. [a]Mean of two independent measurements; [b]Obtained by computer-fitting of survival curves; [c]Spheroids irradiated post-trypsinisation.

treatment may be related to a cell cycle effect. There is an increase in the proportion of $G_1$-like cells with increasing WiDr culture age from 68 (day 6 cultures) to 78% (day 12) (15% increase (West *et al.*, 1988)). It has been shown that, for some cell lines, $G_1$ cells are the most resistant of cells in the cell cycle to photodynamic treatment (Christensen *et al.*, 1981). It is possible that WiDr cells show a variable cell cycle response with $G_1$ resistance and that the increase in proportion of $G_1$ cells in plateau-phase cultures accounts for resistance to PDT. This is supported by the similarity in drug uptake between exponentially growing and plateau-phase cells (Table I). In contrast to the results of Christensen *et al.* (1981), Gomer & Smith (1980) showed no cell cycle response, further supporting the notion of cell line dependent, growth phase related differences.

Although the presence of oxygen is essential for photosensitiser-induced light cytotoxicity (Moan & Sommer, 1985), the size-dependent spheroid resistance seen here may not be related to the presence of hypoxia. For small spheroids there was no difference in photodynamic sensitivity when light irradiations were carried out both before and after trypsinisation to single cell suspensions. This indicates that there was no induced hypoxia as a result of static culture conditions (using static culture conditions for irradiation with sparsely ionising radiation can lead to an increase in the level of hypoxia in spheroids (Durand, 1980)). Secondly, irradiations were carried out in fresh medium and at room temperature, both of which are known to lead to spheroid reoxygenation (e.g. Franko & Koch, 1983). Thirdly, WiDr spheroids, up to 500 $\mu$m in diameter, do not contain central necrosis or radiobiological hypoxia, i.e. there is no hypoxic fraction on the radiation survival curve for 500 $\mu$m spheroids (West, unpublished data). The induction of a hypoxic region in the larger spheroids during light exposure cannot be totally ruled out because of the difference in sensitivity for spheroids irradiated pre- and post-trypsinisation. However, this may be due also to inefficient light penetration through spheroids. The depth of penetration of light in tissue has been described as being 200 $\mu$m at 400 nm and 1 mm at 700 nm (Eichler *et al.*, 1977). A broad spectrum light source was used (West & Moore, 1988) and absorption by Photofrin II of shorter, less penetrating wavelengths (350–550 nm) dominates its cytotoxic effect (West & Moore, 1989).

The question of interest is what causes the marked spheroid size dependent resistance? The small resistance of 100 $\mu$m diameter spheroids may be related to a cell contact effect and there is some evidence that this can occur in monolayer cultures (Christensen & Moan, 1980). The resistance of plateau-phase cells and reduced uptake may be involved also. However, although these may account for the resistance of the smaller spheroid sizes (100, 250 $\mu$m), because of the magnitude of the effect, other factors must be involved in the resistance of large spheroids.

There is some indication that heterogeneity of drug uptake may be important. The majority of *in vitro* photodynamic survival curves have broad and clearly defined shoulders similar to that illustrated in Figure 1 (see also Gomer & Smith, 1980). Spheroids, 100 and 250 $\mu$m in diameter, gave similar shaped curves while the curve for 500 $\mu$m diameter spheroids was a straight line (Figure 3d). It is interesting to compare this spheroid survival curve with that for exponentially growing cells exposed to 1 $\mu g\,ml^{-1}$ drug (Figure 4). Comparison of drug uptake showed that cells exposed to 10-fold less drug accumulated 10-fold less drug (West, unpublished data) and were 10-fold less sensitive (taken from a ratio of $D_0$s (West & Moore, 1988)) Spheroids of 500 $\mu$m diameter took up 3-fold less drug but were 22-fold less sensitive than exponentially growing monolayers. The straight line of the 500 $\mu$m spheroid 'curve' and the shallower slope than would be expected on the basis of reduced average drug uptake suggest an increase in heterogeneity. It is possible that there is a wide spectrum of intracellular drug levels following the exposure of large spheroids to Photofrin II. A compilation of a number of PDT survival curves for different drug-exposed cell populations would produce a survival curve with a shallower slope than each of the composite curves (see Dutreix *et al.*, 1988) and a curve without a shoulder.

A drug penetration problem is the obvious explanation and our initial expectation was of a gradient in drug concentration from the peripheral to the central region. This has been demonstrated in spheroids exposed to the chemotherapeutic agent adriamycin (Sutherland *et al.*, 1979). However, preliminary studies of fluorescence micrographs of centrally cut frozen sections of Photofrin II-exposed spheroids suggest good overall penetration but a patchy distribution between cells with some fluorescing brightly and others dimly regardless of position across the spheroid (West, unpublished data). In addition, there is no tail to the survival curve for spheroids irradiated post-trypsinisation which would be seen if there was a drug-free sub-population of cells.

If drug penetration is not thought to be the cause of heterogeneity of drug uptake within spheroids, what is? It could be related to a cell contact effect. The degree of cell coupling has been shown to decrease as a function of spheroid age and size (Dertinger *et al.*, 1982). Alternatively, or in addition, decreased drug uptake in some cells of spheroids may be related to membrane changes that might occur with increasing spheroid size. Serum lipoproteins have a high affinity for haematoporphyrins and drug uptake may be via receptor-mediated endocytosis of complexes of porphyrin with low density lipoprotein (LDL; see Barel *et al.* (1986) for discussion). There may be a decrease in the number of LDL receptors in some cells when grown in close contact, leading to an increase in the heterogeneity of drug intake.

In conclusion a marked spheroid size dependent resistance to photodynamic treatment has been demonstrated. This

**Figure 4** A comparison of the survival curves for exponentially growing WiDr cells exposed to 10 (from Figure 1) and 1 (from West & Moore, 1988) $\mu g\,ml^{-1}$ and 500 $\mu$m diameter spheroids (from Figure 3c) exposed to 10 $\mu g\,ml^{-1}$ Photofrin II followed by irradiation with light.

resistance cannot be explained simply in terms of hypoxia, reduced drug uptake or the limited penetration of Photofrin II into spheroids. There is evidence to suggest that heterogeneity of intracellular drug levels between cells is important. This may be mediated via changes in the degree of cell contact between cells or in the membrane composition of cells when grown as three-dimensional multicellular spheroids.

Drs J.V. Moore and J.H. Hendry for discussions, Miss S.J. Morrissey for typing the manuscript, and the support of the Cancer Research Campaign (UK) are gratefully acknowledged.

### References

BAREL, A., PERIN, A., ROMANDINI, P., PAGNAN, A. & BIFFANTI, S. (1986). Role of high-, low- and very low-density lipoproteins in the transport and tumour-delivery of hematoporphyrin *in vivo*. *Cancer Lett.*, **32**, 145.

BEN-HUR, E., GREEN, M., PRAGER, A., KOL, R. & RODENTHAL, I. (1987). Phthalocyanine photosensitization of mammalian cells: biochemical and ultrastructural effects. *Photochem. Photobiol.*, **46**, 651.

CHRISTENSEN, T., FEREN, K., MOAN, J. & PETTERSEN, E. (1981). Photodynamic effects of haematoporphyrin derivative on synchronised and asynchronous cells of different origins. *Br. J. Cancer*, **44**, 717.

CHRISTENSEN, T. & MOAN, J. (1980). Photodynamic effect of hematoporphyrin (HP) on cells cultivated *in vitro*. In *Lasers in Photomedicine and Photobiology*, Protesi, R. & Sacchi, C.A. (eds) p. 87. Springer-Verlag: Berlin.

CHRISTENSEN, T., MOAN, J., SANDQUIST, T. & SMEDSHAMMER, L. (1984). Multicellular spheroids as an *in vitro* model system for phororadiation therapy in the presence of HpD. In *Porphyrin Localization and Treatment of Tumours*, Doiron, D.R. & Gomer, C.J. (eds) p. 381. Alan R. Liss: New York.

DERTINGER, H., HINZ, G. & JAKOBS, K.H. (1982). Intercellular communication, three-dimensional cell contact and radiosensitivity. *Biophys. Struct. Mech.*, **9**, 89.

DURAND, R.E. (1980). Variable radiobiological responses of spheroids. *Radiat. Res.*, **81**, 85.

DURAND, R.E. (1981). Flow cytometry studies of intracellular adriamycin in multicell spheroids *in vitro*. *Cancer Res.*, **41**, 3495.

DURAND, R.E. & SUTHERLAND, R.M. (1972). Effects of intercellular contact on repair of radiation damage. *Exp. Cell. Res.*, **71**, 75.

DUTREIX, J., TUBIANA, M. & DUTREIX, A. (1988). An approach to the interpretation of clinical data on the control probability-dose relationship. *Radiother. Oncol.*, **11**, 239.

EICHLER, J., KNOF, J. & LENZ, H. (1977). Measurements on the depth of penetration of light (0.35–1.0 $\mu$m) in tissue. *Radiat. Envir. Biophys.*, **14**, 239.

FRANKO, A.J. & KOCH, C.J. (1983). The radiation response of hypoxic cells in EMT6 spheroids in suspension culture does model data from EMT6 tumours. *Radiat. Res.*, **96**, 497.

GILBERT, C.W. (1969). Computer programmes for fitting Puck and probit survival curves. *Int. J. Radiat. Biol.*, **16**, 323.

GOMER, C.J. & SMITH, D.M. (1980). Photoinactivation of Chinese hamster cells by hematoporphyrin derivative and red light. *Photochem. Photobiol.*, **32**, 341.

HAHN, G.M. & LITTLE, J.B. (1972). Plateau-phase cultures of mammalian cells. *Curr. Topics Radiat. Res.*, **8**, 39.

MOAN, J. & SOMMER, S. (1985). Oxygen dependence of the photosensitizing effect of hematoporphyrin derivative in NHIK 3025 cells. *Cancer Res.*, **45**, 1608.

MOORE, J.V., KEENE, J. & LAND, E.J. (1986). Dose-relationships for photodynamic injury to murine skin. *Br. J. Radiol.*, **59**, 257.

PANTELIDES, M.L., MOORE, J.V. & BLACKLOCK, N.J. (1989). A comparison of serum kinetics and tissue distribution of Photofrin II following intravenous and interperitoneal injection in the mouse. *Photochem. Photobiol.*, **41**, 67.

SUTHERLAND, R.M., EDDY, H.A., BAREHAM, B., REICH, K. & VANANTWERP, D. (1979). Resistance to adriamycin in multicellular spheroids. *Int. J. Radiat. Oncol.*, **5**, 1225.

SUTHERLAND, R.M., McCREDIE, J.A. & KRUUV, J. (1970). A multicomponent radiation survival curve using an *in vitro* tumour model. *Int. J. Radiat. Biol.*, **18**, 491.

WEST, C.M.L., KENG, P.C. & SUTHERLAND, R.M. (1988). Growth phase related variation in the radiation sensitivity of human colon adenocarcinoma cells. *Int. J. Radiat. Onc. Biol. Phys.*, **14**, 1213.

WEST, C.M.L. & MOORE, J.V. (1988). Cell survival characteristics of a human colon adenocarcinoma cell line after photodynamic treatment: a comparison of Photofrin II and TPPS. *Int. J. Radiat. Biol.*, **54**, 62.

WEST, C.M.L. & MOORE, J.V. (1989). The photodynamic effects of Photofrin II, hematoporphyrin derivative, hematoporphyrin, and tetrasodium-meso-tetra(4-sulfonatophenyl)porphine *in vitro*: clonogenic cell survival and drug uptake studies. *Photochem. Photobiol.*, **49**, 169.

WEST, C.M.L., SANDHU, R.R. & STRATFORD, I.J. (1984). The radiation response of V79 and human tumour multicellular spheroids-cell survival and growth delay studies. *Br. J. Cancer*, **50**, 143.

WEST, C.M.L. & STRATFORD, I.J. (1987). A comparison of adriamycin and mAMSA II. Studies with V79 and human tumour multicellular spheroids. *Cancer Chemother. Pharmacol.*, **20**, 109.

WEST, C.M.L. & SUTHERLAND, R.M. (1987). The radiation response of a human colon adenocarcinoma grown in monolayer, as spheroids, and in nude mice. *Radiat. Res.*, **112**, 105.